# Validating MOSPA questionnaire for measuring physical activity in Pakistani women

**DOI:** 10.1186/1475-2891-5-18

**Published:** 2006-08-10

**Authors:** Romaina Iqbal, Ghazala Rafique, Salma Badruddin, Rahat Qureshi, Katherine Gray-Donald

**Affiliations:** 1 Population Health Research Institute, McMaster University, 237 Barton East, Hamilton ON L8L 2X2, Canada; 2 Department of Community Health Sciences & Obstetrics and Gynecology, Aga Khan University, Stadium Road P.O. Box 3500, Karachi 74800, Pakistan; 3 School of Dietetics and Human Nutrition, McGill University, 21, 111 Lakeshore Road, Ste-Anne-de-Bellevue, QC H9X 3V9, Canada

## Abstract

**Background:**

Precise measurements of activity at a population level are important for monitoring trends and evaluating health promotion strategies. Few studies have assessed the measurement of physical activity in developing countries. The aim of this study was to validate the MOSPA (Monica Optional Study of Physical Activity) questionnaire which was developed for the WHO-Monitoring trends and determinants of cardiovasculr disease (MONICA) study sites.

**Methods:**

The MOSPA questionnaire assesses energy expendtiture (EE) related to physical activity (employment, household work, transportation, and leisure time) over a one year period. This questionnaire has been described in the manuscript as the long term (LT) questionnaire. An adapted short term (ST) 5 day questionnaire was developed to assess convergent validity. Questionnaire data were compared with physical activity EE estimates from a Caltrac accelerometer and with body composition measures (height, weight and bioelectrical impedance) in 50 women from the Aga Khan University (AKU) hospital antenatal clinics, Pakistan. Other forms of EE i.e. resting EE and thermic effect of food were not assessd in this study.

**Results:**

Subjects were aged 26 ± 3.8 years and were 16.1 ± 6.7 weeks pregnant. Their average weight was 58.8 ± 10.7 Kg. The average EE/day assessed by the Caltrac accelerometer, was 224 kcal and by MOSPA LT questionnaire it was 404 kcal. The questionnaires and Caltrac data were reasonably well correlated: r = 0.51 and r = 0.60 (P < 0.01) for LT and ST questionnaires respectively. Energy expenditure from questionnaire data was not correlated with body composition measures.

**Conclusion:**

The MOSPA questionnaire is useful in assessing physical activity levels in a sedentary population over a one year period.

## Background

Physical inactivity, diet and other risk factors contribute significantly to the global burden of chronic diseases such as obesity, diabetes, heart disease, stroke, and breast and colon cancer [1]. Over one million deaths annually can be attributed to physical inactivity alone on a worldwide basis [1]. In the developing world, urban areas have higher prevalence rates of chronic diseases than rural areas [2]. One of the reasons suggested for this higher prevalence of inactivity in the urban areas is a sedentary life-style characterized by less physically demanding work [3]. There are a growing number of studies monitoring differences in physical activity across regions and changes over time, [4-6], however, there are few published studies validating measures of self-reported physical activity in developing countries.

Several review papers [7,8] address the reliability and validity of self-reported physical activity questionnaires, however, most of these validation studies were carried out in industrialized country settings where physical activity patterns may be quite different from those in developing countries in terms of leisure activities, transportation and type of work. Three self-report measures validated in developing countries could be found. The sub-Saharan Africa Activity Questionnaire (SSAAQ) conducted in Cameroon showed good agreement with accelerometer measures (r = 0.60–0.74) in an active young population [9]. The International Physical Activity Questionnaire (IPAQ), validated against accelerometers, in 12 countries including South Africa and Guatemala in urban as well as rural populations showed similar agreement in developing countries [10]. The Indian Physical Activity Questionnaire was validated using energy balance, i.e. reported energy intake vs. EE, and showed weak overall correlations r = 0.30 on average with their questionnaire [11].

This study was undertaken to validate a self-reported physical activity questionnaire in a sedentary, urban living population in a developing country, Pakistan. The specific objective of our study was to assess the validity of a WHO administered physical activity questionnaire using Caltrac accelerometers (Muscle Dynamics, Torrance, CA) and body composition measures.

## Methods

This study is part of a larger investigation of the predictors of gestational diabetes in Pakistani women. A sub-sample from the main cohort was invited to participate in this study. The inclusion criteria were women attending an antenatal clinic at AKU Hospital, Pakistan who were less than 24 weeks pregnant. A total of 65 subjects consented to participate in the study. Of this group, 2 withdrew because of family concerns about the safety of Caltrac, 2 lost their pregnancy; 11 did not complete the protocol (could not be reached, did not wear Caltrac regularly, battery failure etc). The study was approved by the ethical review boards of AKU, Pakistan and McGill University, Canada. Informed consent was obtained from all study participants.

Subjects were asked to wear a Caltrac accelerometer at all times for five consecutive days that included week as well as weekend days. The apparatus was not worn while sleeping or bathing. None of the subjects were involved in any water sport activities. On completion of the study, i.e. 120 hours, participants were contacted by the telephone and asked to report the reading on the Caltrac lithium screen. At this time subjects also completed a 5 day physical activity questionnaire. Most of the subjects (n = 35) recruited for this validation study had provided information on physical activity long term (LT), before beginning this study. For subjects (n = 15) who had not done so, the LT questionnaire was conducted by telephone after the Caltrac monitoring period.

### Questionnaire

The physical activity questionnaire used for this study was the MONICA Optional Study of Physical Activity (MOSPA) questionnaire [12] developed by the Centers for Disease Control and Prevention, USA (CDC). This questionnaire captures physical activity for a period of one year. A modified version of the questionnaire was also developed to capture activity during the five days during which the validation was done by changing the reference times. In this paper, the one year questionnaire is referred to as the long term questionnaire (LT) and the adapted questionnaire developed to measure concurrent activity during the five days on which the subjects wore the Caltrac machine as the short term questionnaire (ST).

The MOSPA questionnaire is an adjunct to the WHO Monitoring Trends and Determinants of Cardiovascular Disease (MONICA) study being conducted in several European countries to assess the risk factors of CVD. This questionnaire measures time and energy spent in a range of physical activities including occupational work, transport related activities, household chores as well as leisure time activity over a one year period. The EE is calculated in metabolic equivalent (MET) scores and can be converted to EE in kcal/week as the final output. This was obtained by multiplying the MET scores for each activity by the duration of the activity. The MET scores for each activity were obtained from the MOSPA MET intensity codes developed by CDC, these codes correspond with the Compendium of Physical Activities developed by Ainsworth et al. [13]. Daily energy expenditure (kcal/day) was obtained by dividing the weekly MET scores by 7. The questionnaires (five day and 1 year reference period) were translated into Urdu (the national language of Pakistan). The translated questionnaires were reviewed by members of the research team to ensure that the translations were appropriate. The questionnaires were pilot tested prior to administration.

### Accelerometer

The accelerometer used in this study was the Caltrac accelerometer (Muscle Dynamics Fitness Network, California, USA). It is a uni-axial motion sensor that can detect body movement and convert it into counts that represents frequency of movement or EE (kcal) related to physical activity, using individual data on weight, height, age and sex. This equipment has been previously used as a direct measure of physical activity for validation of self-reported measures [14,15]. It is worn on the waist and captures movement of the lower body.

### Body composition measures

As an indirect means of assessing the validity of the questionnaire, body composition was measured using the Tanita Body composition analyzer TBF 300 A, (Tanita Corp., Tokyo, Japan). The body composition analyzer works on the principle of bio-electrical impedance which measures the flow of a small current through the body, (foot-to-foot in this case) and different tissues of the body conduct the current differently reflecting that in differences in impedance values. Taking into consideration the age, sex, weight and height of each subject, total body water, fat mass, fat percentage and fat free mass are calculated by the analyser using an inbuilt equation. These measures were available for 37 of the 50 women enrolled.

### Statistical methods

The (EE) estimates related to physical activity from both the questionnaires and the Caltrac readings were not normally distributed thus Spearman's rank order correlation was performed to examine correlations among the EE and body composition measures. Repeated measures analysis using Greenhouse-Geisser adjustments for violations of sphericity was carried out to estimate differences between the three EE measures (LT and ST questionnaires and Caltrac). For post-hoc analysis, Bonferroni corrections were used. Data were analyzed using SAS version 8.2 statistical software.

## Results

Fifty subjects completed the study. The women were on average 25.95 ± 3.84 years of age with a BMI of 23.20 ± 4.30 kg/m^2 ^and fat percentage of 26.48 ± 7.23. Forty percent of the subjects were university educated (Table 1). During the course of the study, 8 of the subjects forgot to wear the Caltrac at some point. These lapses averaged 52.5 ± 37.7 minutes (mean ± SD) over the entire study period of five days. Energy expenditure (EE) as measured by Caltrac was not different between subjects who forgot to wear Caltrac versus the subjects reporting complete adherence to the protocol.

**Table 1:** Description of Pregnant Pakistani Women Enrolled in the Validation Study (n = 50)

**Characteristics**	**Mean**	**SD**
Weight (kg)	58.84	10.72
Height (cm)	159.45	6.38
BMI (kg/m^2^)	23.20	4.30
Age (years)	25.95	3.84
Gestational Age at the time of Caltrac study (weeks)	16.14	6.74
Fat (%)*	26.48	7.23
Fat Mass (kg)*	15.74	6.65
Fat Free Mass (kg)*	41.30	3.14
Educational status (% university graduates)	40	-
Employment status (% employed)	32	-

* Data available for 37 subjects

In order to measure the extent to which individual measures of physical activity from MOSPA questionnaires agree with the criterion measure, a correlation matrix for the direct measure of physical activity i.e. the Caltrac activity EE score as well as the indirect measures i.e. the body composition measures is provided (Table 2). Both the questionnaires, LT and ST, were positively correlated with the Caltrac physical activity values (r = 0.51 and 0.60, respectively). None of the body composition indices correlated with any of the three measures of EE with the exception of the BMI and the Caltrac values. Since body weight is part of both the calculation of EE estimated by the Caltrac and BMI, some correlation is to be expected.

**Table 2:** Rank Order Correlations for Physical Activity Measures from Questionnaires with Accelerometer and Body Composition Measures

	**Spearman's Rank Order Correlations**				
**MOSPA QUESTIONNAIRE**	**Caltrac Activity (kcal)**	**Body Fat (%)**	**Fat Mass (kg)**	**Fat Free Mass (kg)**	**BMI (kg/m^2^)**

**STQ** Questionnaire (kcal)	0.60**	0.19	0.24	0.09	0.19
**LTQ** Questionnaire (kcal)	0.51**	0.15	0.14	0.17	0.25
**Caltrac** Caltrac activity (kcal)	-	0.03	0.06	0.20	0.38**

** P < 0.001 level

The mean EE from activity from the MOSPA LT was 403 ± 530 kcal/d. The mean for ST questionnaires was 306 ± 370 kcal/day. The Caltrac estimated less activity at 224 ± 94 kcal/day. The means of the ST questionnaire was not different from the Caltrac energy estimate, however the mean Caltrac energy estimate and the LT questionnaire (activity in past year) were different from each other (P < 0.05) in repeated measures ANOVA.

The MOSPA questionnaire measures reported physical activity in 4 broad categories; work, household chores, leisure and transportation. Based on the analysis of the MOSPA (LT), 16 women (32%) were involved in some kind of occupational work and the average EE calculated for work from the questionnaire was 866 kcal/day. This resulted from work related activity of 5.72 hours/day. Other reported activities, such as household chores or leisure time activities, done by more women, provided much lower levels of EE and were done for much shorter periods of time (Table 3).

**Table 3:** Breakdown of Average Reported Energy Expenditure and Time Spent in Various Activities for Pakistani Women

**Activities**	**n Reporting activity**	**EE (kcal)/day**		**Time (Minutes)/day**	
		**Mean**	**SD**	**Mean**	**SD**
**Work**	16	866	397	343.8	121.2
**Transportation**	17	36	16	8.9	3.9
**Household chores**	24	124	123	56.9	45.3
**Leisure time activities**	31	89	124	25.7	23.6
**Total Activity**	50	404	530	127.3	174.1

Owing to the high EE reported on the questionnaire by the women who worked outside the home and the possibility that this factor had an important impact on the correlations found, a stratified analysis was done to examine the working and the non-working women with respect to the agreement of Caltrac readings with the MOSPA (LT and ST). For the employed women (n = 16) MOSPA ST correlated with the Caltrac readings r = 0.62 (P < 0.05) but MOSPA (LT) was not significantly correlated with Caltrac, r = 0.45 (P = 0.07) but the statistical power is low in this small subset. In the non-working group (n = 34) both MOSPA (LT and ST) correlated with Caltrac, r = 0.47; P < 0.005 and r = 0.60 and P < 0.001 respectively.

On examination of the subset of working women, EE for physical activity of 1078 kcal/day was reported on the LT questionnaire, but the Caltrac value was only 263 kcal. This overestimation was similar in the ST questionnaire. This is a large over estimation by the MOSPA with regards to EE. In contrast, among those not employed the MOSPA (LT) did not capture all activities as the MOSPA (LT) reported a total expenditure of only 86.kcal/day whereas the Caltrac recorded 205 kcal/day (P < 0.001). Findings for the ST questionnaire were similar. MOSPA thus tends to overestimate work activity and underestimate other activity (mainly household) in this setting.

The MOSPA questionnaire also has a self-rating question for physical activity assessment with 4 categories; 1 indicating the least and 4 the most physical activity. The figure shows the self-categorization ratings from the MOSPA (LT) versus the Caltrac physical activity scores for subjects in each category. None of the subjects reported being involved in very vigorous activity and only one person reported moderate physical activity while 32 subjects reported no activity and 17 thought that they were involved in light activity on most of the week days. A comparison between groups 1 and 2 (no activity and light activity) showed a 100 kcal difference (P < 0.001) in kcal by the Caltrac measure.

## Discussion

Despite very low levels of physical activity in this population of young women, the total EE from the ST and LT questionnaires correlated reasonably well with the Caltrac confirming that the questionnaires can assess physical activity levels in even a sedentary urban population. The MOSPA questionnaire is easy to administer and gives a valid measure of activity levels overall, however, questionnaire items to detect work related activities overestimated activity and some low EE activities by women such as caring for self and others are not picked up by the questionnaire.

The absolute EE values, using both the questionnaires as well as the Caltrac, were very low. This is similar to levels of physical activity in Filipino youth where the mean EE as measured by the Caltrac was 271 kcal ± 105.4 kcal/day [16]. In a cross sectional survey conducted in urban India, 49.5% of the population did not engage in any leisure time physical activity as assessed by an interview and another 5.7% performed physical activity irregularly, indicating that the level of leisure time related physical activity is very low in urban, populations in the region [17]. Leisure activity represented a small portion of the activity in our sample as well.

Certain limitations to our validation study need to be recognized. The women studied were on average 16 weeks pregnant which may limit the ability to generalize these results. However, the ST questionnaire reflects their actual activities at the time of the Caltrac measure and the LT questionnaire reflected their usual activities in the last year.

The mean EE, based on LT and ST questionnaires is higher than that from the Caltrac accelerometer; several studies have reported similar findings when using Caltrac accelerometers [8]. This may be because uni-axial Caltrac cannot measure movement in the horizontal plane or because of overestimation of activity on the questionnaire by the subject or the assumptions of the MET scores used to assess total EE. This overestimation seems to be pronounced for the work related activities in this questionnaire.

In the only other study to date validating the MOSPA questionnaire, the criterion measures used were BMI, body composition, and peak oxygen uptake values on an exercise test [18]. The subjects of their study were physical activity instructors or former athletes. A positive correlation of r = 0.53 was observed between the questionnaire and lean body mass. In contrast, our study indicated there were no significant correlations between LT and body composition measures. We speculate that the reason for this lack of correlation is that our study population, as a whole, was not active enough to develop differences in body composition related to activity. Another reason could be an alteration in body composition due to pregnancy but this is unlikely to be substantial given their early stage in pregnancy. The lack of association with biological parameters in our study is unlikely due to a lack of statistical power as our sample size offered power of 0.80 to detect a significant correlation of r = 0.38 as statistically significant.

In a study examining the construct validity of a physical activity questionnaire in the Netherlands, work related activities, sports activities and leisure time activities (other than sports) were the three loading factors for habitual physical activity assessment [19]. In contrast, studies done in South East Asia suggest that often the major contributor to EE are everyday tasks and walking to work/school rather than the leisure time activity, observed in the West [16]. Our study also confirms this finding, as there was a 30% difference based on the Caltrac values between those working outside the home vs. not employed, but the values for EE were low in both groups. One of the probable reasons the questionnaire EE under reported activity for women involved in household work is its inability to assess particular elements such as care giving activities for the elderly and/or child care. Similar findings have also been reported in a Canadian Aboriginal population study where housework was the principal physical activity reported [20].

In a review of seven different types of self-reported activity measures used in industrialized countries, it was observed that questionnaires that recalled activity for a shorter duration, such as past week, had validity correlations of 0.5 with Caltrac accelerometers compared to correlations ranging between 0.14 and 0.36 for longer term, physical activity questionnaires [8]. Although our findings follow the same pattern, the correlations found among our sample of Pakistani women were higher than the correlations reported in the literature on adult participants. Although the correlation of the ST physical activity questionnaire is higher than the LT questionnaire, we do not suggest the use of the ST questionnaire to measure physical activity because of a lack of consistency in activity over weeks, variations due to seasonality, acute illness or other reasons for variability over time as have been reported by others [21].

Comparing our findings to other reports of physical activity questionnaires which were validated using accelerometers in developing countries, we found similar correlations. The IPAQ [10] conducted in 14 countries including 2 developing countries, South Africa and Guatemala reported correlations of 0.46 and 0.61 respectively in these two countries for their urban population and these were far higher correlations than in the industrialized nations participating in the study. Similarly in a validation study in sub-Saharan Africa [9] correlations of 0.60 and 0.74 for females and males respectively were observed in a very active population.

The last item in the MOSPA LT questionnaire is a global question asking subjects to categorize themselves into 4 categories. We feel that the subjects are quite cognizant of their generally low levels of physical activity as they accurately classified themselves as being sedentary. The difference in Caltrac values between the two lowest levels of activity was significant. Such self-reporting scales are not, however, always successful.

We have shown that overall EE levels related to physical activity are very low in our study population which could be a significant predictor of rising chronic disease prevalence in the region. We have demonstrated that MOSPA physical activity questionnaire is able to assess physical activity levels adequately in a sedentary population. As the questionnaire tends to overestimate activity related to work, and not capture some household movement, some refinements in this assessment measure may enhance the precision of the questionnaire.

## Competing interests

The author(s) declare that they have no competing interests.

## Authors' contributions

RI designed the study, collected and analysed the data as well as wrote the manuscript. GR, SB and RQ helped in data collection and provided critical review of the manuscript. KGD supervised the development of the study protocol, analysis and helped to draft and revise the manuscript. All authors read and approved the final manuscript.

**Figure 1:**
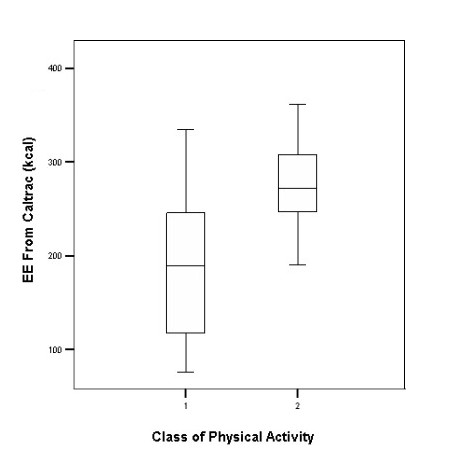
Self – categorization of Pakistani women into different self- reported physical activity levels.
